# Recent Positive Selection in Genes of the Mammalian Epidermal Differentiation Complex Locus

**DOI:** 10.3389/fgene.2016.00227

**Published:** 2017-01-10

**Authors:** Zane A. Goodwin, Cristina de Guzman Strong

**Affiliations:** Division of Dermatology, Department of Internal Medicine, Center for Pharmacogenomics and Center for the Study of Itch, Washington University School of Medicine, St. LouisMO, USA

**Keywords:** positive selection, evolution, skin, epidermis, barrier, epidermal differentiation complex

## Abstract

The epidermal differentiation complex (EDC) is the most rapidly evolving locus in the human genome compared to that of the chimpanzee. Yet the EDC genes that are undergoing positive selection across mammals and in humans are not known. We sought to identify the positively selected genetic variants and determine the evolutionary events of the EDC using mammalian-wide and clade-specific branch- and branch-site likelihood ratio tests and a genetic algorithm (GA) branch test. Significant non-synonymous substitutions were found in *filaggrin, SPRR4, LELP1*, and *S100A2* genes across 14 mammals. By contrast, we identified recent positive selection in *SPRR4* in primates. Additionally, the GA branch test discovered lineage-specific evolution for distinct EDC genes occurring in each of the nodes in the 14-mammal phylogenetic tree. Multiple instances of positive selection for *FLG, TCHHL1, SPRR4, LELP1*, and *S100A2* were noted among the primate branch nodes. Branch-site likelihood ratio tests further revealed positive selection in specific sites in *SPRR4, LELP1, filaggrin*, and *repetin* across 14 mammals. However, in addition to continuous evolution of *SPRR4*, site-specific positive selection was also found in *S100A11, KPRP, SPRR1A, S100A7L2*, and *S100A3* in primates and *filaggrin, filaggrin2*, and *S100A8* in great apes. Very recent human positive selection was identified in the *filaggrin2* L41 site that was present in Neanderthal. Together, our results identifying recent positive selection in distinct EDC genes reveal an underappreciated evolution of epidermal skin barrier function in primates and humans.

## Introduction

The evolution of modern humans (*Homo sapiens sapiens*) is driven by ongoing adaptations to local environments and niches (Jeong and Di Rienzo, 2014). Insights into the biological pathways and genes underlying human evolution have been identified from early pairwise comparisons between the coding sequences of human and its closest primate relative, the chimpanzee (*Pan troglodytes*) (Clark et al., 2003). Additional studies discovered positive selection for gene variants involved in sensory perception, amino acid catabolism, host defense/immunity, reproduction, hair follicle development, and skin pigmentation in human evolution (Nielsen et al., 2005; Voight et al., 2006; Sabeti et al., 2007; Kosiol et al., 2008). The inclusion of additional mammalian genomes provided higher resolution into specific biological function, including innate complement immunity and taste perception (Bakewell et al., 2007; Kosiol et al., 2008).

A direct comparison to the complete genome of the chimpanzee enabled a closer investigation of more recent evolution in the human genome (Chimpanzee Sequencing and Analysis Consortium, 2005). From this study, the epidermal differentiation complex (EDC) locus was identified as the most rapidly evolving locus in the human genome among other loci involved in immunity, perception, and epithelia (Chimpanzee Sequencing and Analysis Consortium, 2005).

The EDC exhibited the highest proportion of amino acid substitutions (*K_A_*/*K_I_* > 1 where *K_A_* and *K_I_* denotes nucleotide substitutions that affect codon and intronic/intergenic nucleotide changes, respectively). This finding provided evidence for positive selection in the EDC. The EDC on human 1q21 spans approximately 1.6 Mb and comprises 65 genes representing four gene families including the Filaggrin (*FLG*)-like or SFTP (S100 fused type protein), Late Cornified Envelope (*LCE*), Small Proline Repeat-Rich (*SPRR*) and S100-domain (*S100*) genes (Mischke et al., 1996; de Guzman Strong et al., 2010). The expression of key genes in the EDC [including filaggrin (*FLG*), loricrin (*LOR*), involucrin (*IVL*), *SPRRs, LCEs*, and *S100A7, A10, A11*] is a hallmark feature of terminally differentiated epidermal cells (or keratinocytes) that comprise the mature, stratified layers of the interfollicular epidermis at the skin surface and found between hair follicles (Candi et al., 2005). EDC proteins including FLG, LOR, IVL, and many SPRRs and LCEs are covalently cross-linked in the formation of the cornified envelope that surrounds the keratinocyte as a single structural unit of the epidermal barrier (Candi et al., 2005). The linearity and synteny of the EDC in both eutherian and metatherian mammals have greatly facilitated a more accurate identification of orthologous EDC genes in other primates and mammals (Mischke et al., 1996; Hardman et al., 1999; Cabral et al., 2001b; Jackson et al., 2005; de Guzman Strong et al., 2010; Henry et al., 2012; Jiang et al., 2014; Strasser et al., 2014).

Early comparative genome-wide scans were successful in identifying olfaction and spermatogenesis in human evolving traits (Clark et al., 2003; Nielsen et al., 2005; Kosiol et al., 2008). However, the EDC had not been implicated despite the inclusion of only a small subset of *S100* annotated genes from the EDC at the time of their analyses. These genomic studies included a range of 3–6 species whose genomes had been sequenced at the time. Furthermore, despite the discovery of the human EDC with the highest amino acid substitutions compared to the chimpanzee, we still do not understand which individual genes and their variants that are under positive selection in the human genome (Chimpanzee Sequencing and Analysis Consortium, 2005). Moreover, we also have a poorer understanding of the evolutionary history of the mammalian EDC that is expressed in the interfollicular epidermis. The inclusion of additional, high-quality mammalian genomes for our study will enable more improved comparative genomic analyses to determine such genes.

Here, we sought to more comprehensively identify the genes that underlie the rapid evolution of the human EDC. We aim to gain a better understanding of the evolution of both mammalian and human interfollicular epidermis. Knowledge of evolving EDC genes is critical toward developing hypotheses that will be tested for skin barrier function in mammals and humans. Ultimately, the knowledge gained from these comparative genomics studies and downstream functional analyses will motivate parallel studies in other tissue types and advance our understanding of mammalian and human evolution.

To identify positively selected EDC genes, we used robust statistical measures from manually curated annotations of EDC genes obtained from a comprehensive set of nine publicly available primate genome builds (Lindblad-Toh et al., 2011) as well as the inclusion of dog, opossum, rat, and mouse genomes to the human genome, totaling 14 genomes. Specifically, we aimed to identify both the genes and the single nucleotide changes responsible for the high non-synonymous substitution ratio observed across the entire EDC and to estimate when each gene underwent positive selection during mammalian and primate evolution.

Our results among the studied genes collectively identified significant mammalian-wide positive selection in *Filaggrin* (*FLG*), *SPRR4, late cornified envelope-like proline-rich 1* (*LELP1*), and *S100A2* in the branch likelihood ratio tests (B-LRTs). Clade-specific B-LRT analyses further identified more recent positive selection in *SPRR4* in primates. Using genetic algorithm (GA)-branch tests, we pinpoint multiple instances of positive selection for *FLG, TCHHL1, SPRR4, LELP1*, and *S100A2* across many nodes of primate origin and discover lineage-specific evolution for distinct EDC genes. We also identified site-specific positive selection in *SPRR4, LELP1, FLG*, and *RPTN* when testing across all 14 mammals using branch site-specific likelihood ratio tests (BS-LRTs). Clade-specific BS-LRTs highlighted recent evolution in *S100A11, KPRP, SPRR1A, S100A7L2*, and *S100A3* (primates) and *FLG* and *FLG2* (great apes). Finally, we determine even more recent site-specific positive selection for L41 in *FLG2* in humans. Thus, our study provides a deeper molecular understanding of the evolution of human and primate skin for epidermal barrier function.

## Materials and Methods

### EDC Ortholog Analyses

**FIGURE 1:**
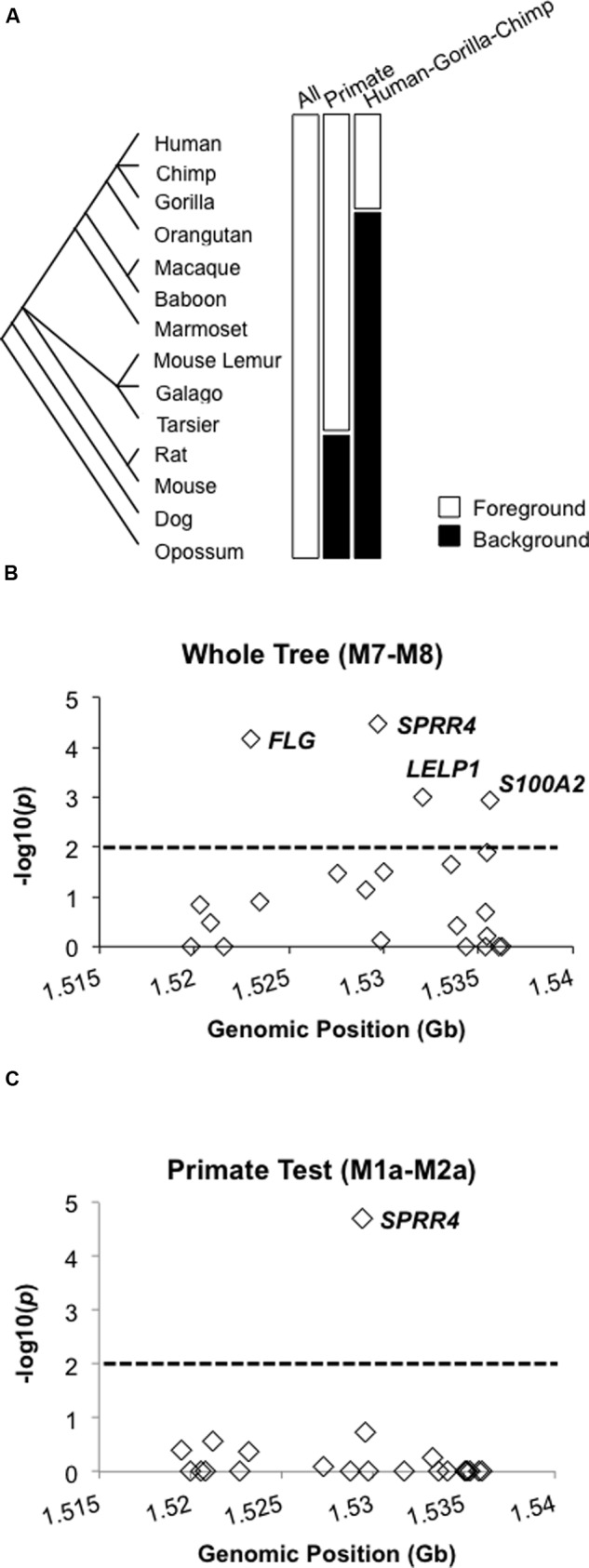
**Positive selection of *FLG, SPRR4, LELP1*, and *S100A2* in 14 mammals and *SPRR4* in primates. (A)** B-LRT conducted for all 14 mammals, primates, and the HGC clades. The following genes were identified for the respective B-LRT: **(B)** *FLG, SPRR4, LELP1*, and *S100A2* (M7–M8 Test) and **(C)** *SPRR4* (primate B-LRT [M1a–M2a test]). Dotted line 1% significance cutoff corresponds to a FDR of 0.05 for this dataset as determined by the Benjamini and Hochberg method. Genes with *p* = 0 were assigned –log10 *p*-values of 0.

Orthologs for each of the human EDC reference genes were downloaded from NCBI and ENSEMBL v. 81 ID (Smedley et al., 2015) using eutils and BioMart, respectively (Yates et al., 2016). For those orthologs which could not be retrieved from the aforementioned method, we manually obtained and annotated the ortholog using either best-hit reciprocal BLAST or the BLAT feature to the respective genome [UCSC genome browser (Speir et al., 2016)]. Direct ortholog comparisons exclude potential biases that can stem from gene flow or recombination when estimating selection (Anisimova et al., 2003; Arenas and Posada, 2010, 2014). Where possible, the longest isoform or ORF [predicted using Geneious v8.1 (Kearse et al., 2012)] for each gene was used for the multiple alignment using MUSCLE (Edgar, 2004). After alignment, codon substitutions were converted to rates whereby *dN* = non-synonymous and *dS* = synonymous substitutions (also known as *K_A_* and *K_S_* or *KN* and *KS*). Positive selection was determined by the significance of the calculated *dN/dS* likelihood ratios using phylogenetic analysis with maximum likelihood (PAML) (Yang, 1998, 2007), specifically branch and branch-site likelihood ratio tests (B-LRT and BS-LRT, respectively). For each B-LRT and BS-LRT that was tested on each gene, the *dN/dS* ratios were tested under the assumptions of two pair-wise statistical models (M1a–M2a and M7–M8). M1a models genetic drift by constraining *dN/dS* values < 1 in comparison to M2a’s assumption of *dN/dS* > 1 for positive selection. In the more sensitive pairwise model that is beta distributed, M7 models genetic drift (0 ≤*dN/dS* ≤ 1) vs. the M8 positive selection model whereby *dN/dS* > 1 (Anisimova et al., 2001; Wong et al., 2004). For each model, raw *dN, dS* and *dN/dS* ratios for all sites and all lineages were estimated using the Nei-Gojobori Method (Nei and Gojobori, 1986). Both B-LRTs and BS-LRTs were performed, with B-LRT testing the entire length of a tested gene and BS-LRT for individual sites for a specific gene. With respect to the mammalian phylogeny of each genome, each B-LRT or BS-LRT was tested across the entire 14-mammal tree (according to Murphy et al., 2001) in accordance with the general consensus in the vertebrate phylogeny community) and in two foreground vs. background clade-specific tests (also **Figure 1**). By definition, as the M7 and M8 model is designed only to test for selection across all branches in a phylogenetic tree, clade-specific LRTs are not possible for the M7–M8 comparison. Each model (M) also computes likelihood values that can be tested for significance using a chi-squared test (df = 2 for the M1a–M2a and M7–M8 comparisons). *p*-values were adjusted to account for both tests across multiple genes and across four different lineages (23 genes in four lineages = 92 hypotheses) using the p.adjust package in R with method = “fdr” according to Benjamini and Hochberg’s method to control for the false discovery rate (Anisimova and Yang, 2007; Bakewell et al., 2007). A *p-*value cutoff (α) of 0.01 corresponds to a FDR of 0.05 for this dataset, hence α = 0.01 is the significance cutoff for the B-LRT. The posterior probabilities (Posterior Prob.) of positive selection for the BS-LRT were calculated using the Bayes’ Empirical Bayes method in PAML for the M1a–M2a and the M7–M8 comparisons as previously described (Yang et al., 2005).

Positive selection for internal branches of the phylogenetic tree were determined by *dN-dS* and estimated using the GA-branch method (Branch-SiteREL) in HyPhy (Pond and Frost, 2005; Pond et al., 2005). A universal genetic code was assumed and the same phylogenetic tree that was used for the PAML tests as previously described above. We allowed the method to automatically decide on model complexity among branches. Both *dN* and *dS* were allowed to vary along branch-site combinations. Internal branch-specific *dN*-*dS* totals were extracted from the data lines labeled “baselineTree” in the “mglocal.fit” output files produced by HyPhy’s GA-branch method.

### Validation of EDC Variants in Neanderthal and Densiova Genomes

Alignments of reads from the Denisovan and Altai Neandertal genome sequencing projects were downloaded from http://cdna.eva.mpg.de/denisova/alignments/ and http://cdna.eva.mpg.de/neandertal/altai/AltaiNeandertal/bam/, respectively. Read count calculations and alignment images were performed using the UCSC genome browser.

## Results

### Evidence of Positive Selection for *FLG, SPRR4, LELP1*, and *S100A2* across Mammals and *SPRR4* in Primates

We sought to accurately determine which of the EDC genes had undergone positive selection in primate and human lineages in the context of mammalian phylogeny. To do this, we utilized comparative alignments among 14 mammalian species genomes. We included the genomes from human (*Homo sapiens*), nine primate species (chimp [*Pan troglodytes*], gorilla [*Gorilla gorilla*], Sumatran orangutan [*Pongo abelii*], macaque [*Macaca mulatta*], baboon [*Papio anubis*], marmoset [*Callithrix jacchus*], mouse lemur [*Microcebus murinus*], galago [*Otolemur garnettii*], tarsier [*Tarsius syrichta*], and four phylogenetically distant mammalian species (rat [*Rattus norvegicus*], mouse [*Mus musculus*], dog [*Canis lupus familiaris*], and opossum [*Monodelphis domestica*]) (**Figure 1A**) (Anisimova et al., 2001). The nine primate species met our selection criteria for the most complete EDC orthology to the human reference among the 15 publicly available primate genomes at the time of our investigation. We define our criteria to be the existence of an ortholog in all 13 mammals for the human reference gene. Based on these criteria, 23 EDC genes met 1:1 orthology to the human reference gene in the 14-mammal dataset. We first identified the genes undergoing positive selection as defined by genes exhibiting greater non-synonymous (*dN)* vs. synonymous (*dS*) substitution ratios (*dN/dS* > 1) and were significant. Positive selection based on the significance of the *dN/dS* ratios (*p* < 0.01 at FDR, 0.05) was determined in which the null hypothesis in favor of neutral evolution was rejected.

Three B-LRT variations were performed to include (1) all 14 mammals and in pair-wise comparisons of the *dN/dS* ratios in the foreground clades of (2) all primates and (3) the human, gorilla, and chimp clade (HGC, representing great apes) to the respective background (remaining) clades (**Figure 1A**). This tiered approach enabled us to determine the point(s) at which an EDC gene underwent positive selection. For the 14-mammal comparisons, we considered pairwise model comparisons between M1a (purifying/negative selection with *dN/dS* < 1) vs. M2a (positive selection) and M7 (beta-distributed *dN/dS*; 0 < *dN/dS* < 1) vs. M8 (positive selection with beta-distributed *dN/dS*) (Supplementary Tables S1 and S2). Across the whole tree and for all sites in the entire alignment in both the M1a–M2a and M7–M8 comparisons, all genes demonstrated *dN/dS* < 1 consistent with purifying (negative) selection (Supplementary Tables S1 and S2). However, when considering individual sites exhibiting *dN/dS* > 1 for each of the orthologous gene sets, the 14-mammal B-LRT in the M1a–M2a comparison identified 12 genes but was not significant (*dN/dS* ratio range, 1.42–24.89; 1–28% of sites) (**Table 1**). By contrast, a total of 16 genes for the 14-mammal B-LRT in the more sensitive M7–M8 model comparison were identified (*dN/dS* ratio range, 1.09–6.11; 0.001–25% of sites) (**Table 1**). Of the 16 genes, four genes in the M7–M8 test were significant (*FLG, SPRR4, LELP1*, and *S100A2*; **Figure 1B**). Together, our 14-mammal B-LRT results identified positive selection for *FLG, SPRR4, LELP1*, and *S100A2* genes across the mammalian lineage.

**Table 1:** Branch likelihood ratio test (B-LRT) results across 14 mammals for each EDC gene that exhibited site –specific proportions with *dN/dS* Ratios > 1 for the M7–M8 comparison.

		All sites			Sites with *dN/dS* > 1			
Branch likelihood ratio test (B-LRT)	Gene	*dN*	*dS*	*dN/dS*	Proportion	*dN/dS*	LRT χ^2^ statistic	*p*
**M7 vs. M8**	*TCHHL1*	0.27	0.52	0.52	0.25	1.39	2.22	3.29 × 10^-1^
	*RPTN*	0.20	0.71	0.30	0.21	2.90	48.74	0.00
	***FLG***	**0.48**	**1.04**	**0.46**	**0.23**	**1.82**	**19.25**	**6.60** ×**10**^-^**^5^**
	*FLG2*	0.08	0.59	0.12	0.12	3.47	4.08	1.30 × 10^-1^
	*KPRP*	0.16	0.49	0.30	0.01	5.01	6.79	3.35 × 10^-2^
	*IVL*	0.41	0.98	0.38	0.46	1.31	5.27	7.17 × 10^-2^
	***SPRR4***	**0.17**	**0.84**	**0.21**	**0.28**	**5.30**	**20.56**	**3.43** ×**10**^-^**^5^**
	*SPRR1A*	0.08	0.51	0.16	0.36	1.09	0.61	7.38 × 10^-1^
	*SPRR3*	0.26	0.91	0.23	0.38	1.24	6.84	3.27 × 10^-2^
	***LELP1***	**0.11**	**0.93**	**0.16**	**0.24**	**3.19**	**13.86**	**9.78** ×**10**^-^**^4^**
	*S100A9*	0.34	0.79	0.42	0.07	2.38	7.64	2.20 × 10^-2^
	*S100A6*	0.05	0.30	0.11	0.03	1.23	3.14	2.08 × 10^-1^
	*S100A5*	0.04	0.53	0.12	1.0E-05	2.87	-1.03 × 10^-3^	0.00
	*S100A4*	0.03	0.39	0.06	0.03	1.59	8.74	1.27 × 10^-2^
	***S100A2***	**0.10**	**0.45**	**0.23**	**0.06**	**1.95**	**13.56**	**1.14** ×**10**^-^**^3^**
	*S100A1*	0.01	0.39	0.02	1.0E-05	6.11	-1.88 × 10^-3^	0.00

*EDC genes are sorted by genomic start position (hg38). Significant genes (*p* < 0.01, based on FDR [false discovery rate]) in bold.*

Clade-specific B-LRT analyses using only M1a and M2a enabled us to determine more recent occurrences of positive selection unique to either primates or great ape clades (each tested as a foreground) vs. background (respective remaining clade) (Supplementary Tables S3 and S4). In the primate-specific B-LRT, we identified eight EDC genes exhibiting regions with *dN/dS* ratios > 1 compared to the background clade (**Table 2**). This was in contrast to many EDC genes exhibiting *dN/dS* < 1 associated with purifying selection (Supplementary Table S3). Of the six genes exhibiting *dN/dS* > 1 in the primate-specific B-LRT, only *SPRR4* was significant (*p* = 2.00 × 10^-5^, *dN/dS* = 4.88) (**Figure 1C**; **Table 2**).

**Table 2:** Branch likelihood ratio test results in the primate foreground clade-specific test for each EDC gene with *dN/dS* Ratios > 1 using the M1a–M2a comparison.

Likelihood ratio test	Gene	% Foreground sites with *dN/dS* > 1	Background Clade (*dN/dS*)	Foreground Clade (*dN/dS*)	LRT χ2 Statistic	LRT *p*-value
**Primate test**	*RPTN*	0.1	0.25	1.44	2.65	2.65 × 10^-1^
	*FLG2*	0.15	0.12	2.54	1.69	4.30 × 10^-1^
	*KPRP*	0.22	0.12	1.15	0.47	7.90 × 10^-1^
	***SPRR4***	**0.16**	**0.04**	**4.88**	**21.64**	**2.00** ×**10**^-^**^5^**
	*SPRR1A*	0.02	0.04	4.93	3.40	1.83 × 10^-1^
	*S100A9*	0.04	0.18	2.67	1.16	5.59 × 10^-1^

*Significant genes (*p* < 0.01, based on FDR [false discovery rate]) in bold.*

When testing for more recent positive selection in the EDC using the great ape HGC as the foreground in the B-LRT (Supplementary Table S4), our analysis identified seven genes exhibiting *dN/dS* ratios > 1. However, none of them were significant. Nevertheless, the observations of the significances of positive selection in *SPRR4* in both the 14-mammal and primate-specific B-LRTs highlight the ongoing evolution of *SPRR4* that extends to primates.

**FIGURE 2:**
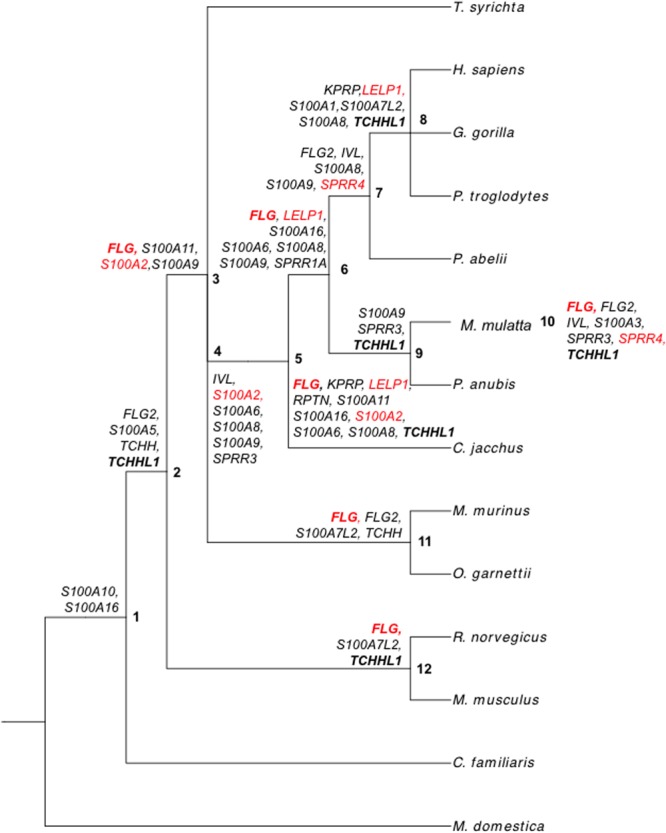
**Positive selection of EDC genes on internal branches of the 14-mammal mammalian phylogeny.** GA-branch test results for all EDC genes. Internal branches (nodes) are labeled with numbers. Genes with *dN-dS* > 0 on internal branches are written next to their respective nodes. Bold text corresponds to genes with *dN-dS* > 0 in at least six nodes. Red text corresponds to genes identified with the B-LRT.

We next sought to further discover where positive selection in the EDC was occurring in our 14-mammal tree. Using the GA-branch method, we identified branch-specific occurrences of positive selection in the EDC in 12 ancestral nodes (**Figure 2**; Supplementary Table S5). At least two genes were found to have undergone positive selection in each of the 12 nodes. All genes were also identified as positively selected in at least one node except for *S100A4*. Both *FLG* and *TCHHL1* were each identified in six different nodes and thus represent the top two genes that underwent positive selection in multiple nodes. Positive selection for *FLG* was found in primate nodes (Nodes 3, 5, 6, 11, and 10) but also in rodents (Node 12). Identification of *TCHHL1* for positive selection was found in primate nodes (Nodes 2, 5, 8, 9, and 10) and in rodents as well (Node 12). As *TCHHL1* was not identified in the B-LRT (observed *dN/dS* = 1), the observations of positive selection for *TCHHL1* in the GA-branch test assert an evolution that is more lineage-specific and parallel. The GA-branch test also revealed that *LELP1, S100A2*, and *SPRR4* (found in B-LRT) underwent multiple rounds of positive selection in the primate lineage. Furthermore, *SPRR4* exhibited *dN*-*dS* > 0 in the primate clade (Nodes 7 and 10), supporting its identification by the M1a–M2a B-LRT. Overall, our GA-branch data further support positive selection for *FLG, LELP1, S100A2*, and *SPRR4* that occurred in multiple nodes in the primate lineage. As well, our results additionally identify evolution for distinct EDC genes in a lineage-specific manner.

### A Majority of Site-Specific Positive Selection Occurred in Conserved Protein Domains of EDC genes

**FIGURE 3:**
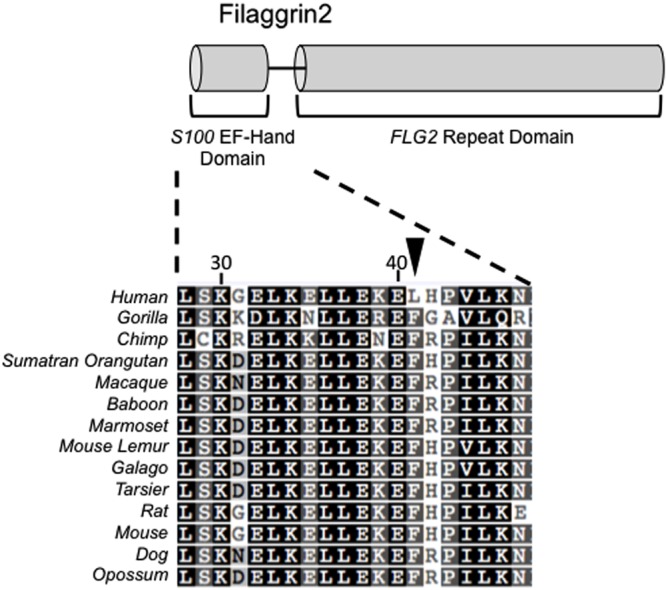
**Human-specific positive selection in the L41 site of *FLG2*.** Arrow highlights alignment of the L41 site of *filaggrin-2* (within the S100 Ca^2+^ binding domain) that was identified by the BS-LRT and specific to the human lineage compared to the mammalian F41 codon. Numbers represent the amino acid (AA) position.

Using the branch-site likelihood ratio test (BS-LRT), we next sought to identify the individual codon substitutions that explain the positive selection in EDC genes. The BS-LRT was performed on 14-mammal alignments for each EDC gene across all species (M1a–M2a and M7–M8) as well as in the pairwise comparisons of the primate and HGC foreground to their respective background clades (with M1a–M2a models’ comparison) as previously described (**Figure 3**). The posterior probability of positive selection (that is, the probability that a given site is in a class of sites with *dN/dS* > 1 [Posterior Prob.], see Materials and Methods) acting on specific codons was subsequently determined for each of the three BS-LRTs. We further determined the locations for the positively selected sites identified by the BS-LRT to gain insight into the functional impact.

The 14-mammal BS-LRT in the M1a–M2a comparison identified evidence for positive selection in codon 60 in the conserved cornifin domain of *SPRR4* (60, human reference position; *dN/dS* = 3.42; Posterior Prob = 0.97) (**Table 3**). Positive selection was also found in the same codon 60 site in *SPRR4* in the 14-mammal BS-LRT (M7–M8 comparison) as well as in sites for *LELP1, FLG*, and *RPTN*. Four sites were found in *LELP1* (*dN/dS* range, 3.33–3.43) and were located within *LELP1*’s conserved cysteine and proline-rich domains. Six sites were found in *FLG (1.51–1.53)*, and 17 sites were all found within the conserved glutamine rich protein domain of *RPTN (2.48–2.56*) (Posterior Prob ≥ 0.95). Six sites (including codon 60 and 5 additional) were determined in *SPRR4* (*dN/dS* range, 5.30–5.35). The codon 60 site was identified in both the M7–M8 and the more stringent M1a–M2a comparison indicating the significance of codon 60 in *SPRR4*’s cornifin domain across the mammalian lineage.

**Table 3:** Branch site-specific likelihood ratio test (BS-LRT) results across 14 mammals for each EDC gene for site-specific positive selection (Posterior Prob ≥ 0.95) in either M1a–M2a or M7–M8 comparisons.

Branch site likelihood ratio test (BS-LRT)	Gene	Reference amino acid (human)	Human position	Amino acid variation	Alignment position	Posterior probability	Site *dN/dS*	Site *dN/dS* SD	Affected domain
**M1a vs. M2a**	*SPRR4*	I	60	I/V/P/A/T/C	87	0.972	3.42	2.04	Cornifin
**M7 vs. M8**	*SPRR4*	G	58	G/K/G/S/Q	85	0.994	5.32	1.83	
		I	60	I/V/P/A/T/C	87	1.00	5.35	1.81	Cornifin
		I	61	I/E/N/P	88	0.991	5.31	1.84	
		C	68	C/V	94	0.989	5.3	1.85	
		Q	72	Q/C/A	124	0.99	5.3	1.84	
		A	73	A/S/Q/D/P	125	0.997	5.34	1.81	
	*LELP1*	K	57	K/P/Q/N	63	0.989	3.39	1.35	Cys/Pro-region
		S	63	S/P/G/M/P/L/F	75	0.999	3.43	1.35	Cys/Pro-region
		K	76	K/C/P	123	0.992	3.41	1.36	Cys/Pro-region
		K	81	K/P/T/S/K	135	0.973	3.33	1.35	Cys/Pro-region
	*FLG*	S	155	S/Q/H/V/A/T/K	171	0.971	1.53	0.23	
		S	180	S/L/A/Q/R	199	0.961	1.52	0.24	
		N	2562	H/R/S/T/Q/K/N	2636	0.966	1.52	0.23	
		F	2567	V/S/T/G/A/S/I	2641	0.960	1.52	0.24	
		Q	2574	E/Q/S/T/P/	2648	0.952	1.51	0.25	
		R	3373	R/H/Q/A/S	3556	0.955	1.51	0.25	
	*RPTN*	R	166	R/Q/T	221	0.965	2.5	0.38	
		H	246	L/Q/C/F/S	305	0.998	2.56	0.25	
		H	258	H/R/Y/C/A	322	0.991	2.55	0.28	
		P	526	P/M/T/S	864	0.964	2.5	0.39	
		M	538	M/T/P/K/S	878	0.966	2.5	0.38	
		K	596	K/S/R/T	945	0.955	2.48	0.41	
		T	604	Q/T/K/R	954	0.993	2.55	0.27	
		L	619	L/S/P/A	969	0.951	2.48	0.43	
		W	679	W/G/R/S	1029	0.956	2.49	0.42	
		S	681	S/L/K	1031	0.976	2.52	0.35	
		W	704	W/Y/G/C/Q	1068	0.974	2.52	0.35	
		H	719	H/R/P/V/C	1090	0.966	2.5	0.38	
		C	726	C/Y/N/W/Q	1097	0.971	2.51	0.36	
		R	768	R/H/Q/H/S	1146	0.954	2.48	0.41	
		R	770	R/D/Q/N/E	1190	0.965	2.5	0.38	
		T	775	T/S/N/R/Q	1195	0.978	2.52	0.34	
		E	779	E/G/K/S/N	1199	0.960	2.49	0.40	

When testing for recent positive selection in the primate clade, we observed site-specific positive selection in six genes (*S100A11* [four sites], *KPRP* [three sites], *SPRR4* [five sites], *SPRR1A* [one site], *S100A7L2* [21 sites], and *S100A3* [two sites]) (all, Posterior Prob ≥ 0.95) (**Table 4**). We next considered the functional impact of these significant substitutions with respect to protein domains. *KPRP* sites did not map within conserved protein domains. However, three out of four sites (codons 69, 72, 73, and 78) in *S100A11* and one out of the two sites (codon 83) in *S100A3* both mapped within the S100 EF-hand domains of the respective proteins. As well, 15 out of the 21 positively selected sites in *S100A7L2* also mapped within an S100 EF-hand domain (**Table 4**). Each EF hand is comprised of two alpha helices separated by a calcium binding domain that imparts calcium signaling for S100 proteins (Santamaria-Kisiel et al., 2006). Observance of the positively selected substitutions occurring within S100 EF hands suggests modulations of either calcium binding or downstream binding of target proteins upon calcium-binding-induced conformational changes to the S100 protein. The P32 site and all five sites (codons 26, 39, 43, 70, and 78) were located within the cornifin domains of *SPRR1A* and *SPRR4*, respectively. Although the codon 60 site in *SPRR4* in its cornifin domain showed significant variation in *dN/dS* across 14 mammals, this same site was not detected in the primate clade test (M1a–M2a) indicating that different sites in *SPRR4* were under positive selection in primates vs. mammals. The cornifin domain found in SPRR proteins is crosslinked by transglutaminase to form the scaffold of the cornified envelope, a structural unit for the epidermal skin barrier (Marvin et al., 1992). The substitutions in the cornifin domains of *SPRR1A* and *SPRR4* suggest modulation of cornified envelope scaffold with an anticipated effect on skin barrier function. Together, we find a majority of the positively selected substitutions within conserved protein domains further highlighting significant evolutionary changes in *S100A11, S100A3, S100A7L2, SPRR1A*, and *SPRR4*.

**Table 4:** Branch site-specific likelihood ratio test results in the primate and HGC foreground clade-specific tests for each EDC gene with site-specific positive selection (Posterior Prob ≥ 0.95) using the M1a–M2a comparison.

Branch site likelihood tatio test (BS-LRT)	Gene	Reference amino acid (human)	Human position	Amino acid variation	Alignment position	Posterior probability	Affected domain
**M1a vs. M2a (primate clade)**	*S100A11*	T	69	T/K/L/I	78	0.99	S100 EF-Hand
		D	72	D/R	81	0.968	S100 EF-Hand
		G	73	G/P	82	0.955	S100 EF-Hand
		S	78	S/Q	87	0.981	
	*KPRP*	C	113	C/R/G/E	204	0.967	
		Y	221	Y/C/R/L	524	0.962	
		R	321	R/H/S/G/C	718	0.964	
	*SPRR4*	P	26	P/A/S	33	0.983	Cornifin
		A	39	A/H/V/P	46	0.987	Cornifin
		K	43	K/P	50	0.965	Cornifin
		A	70	A/K/V/T	122	0.988	Cornifin
		Q	78	Q/V	130	0.983	Cornifin
	*SPRR1A*	P	32	P/T/I	97	0.963	Cornifin
	*S100A7L2*	L	16	L/Q/P/E/Q	74	0.995	
		G	17	G/A/R/P/V/M/I	75	0.954	
		L	22	L/I/V/M/F	80	0.987	S100 EF-Hand
		A	26	A/D/T/N/D/H	84	0.999	S100 EF-Hand
		M	27	M/L/I/L/C	85	0.994	S100 EF-Hand
		S	32	S/T/V/A	90	0.984	S100 EF-Hand
		D	34	D/R/S/P	95	0.999	S100 EF-Hand
		M	40	M/K/V/E/L	101	0.985	S100 EF-Hand
		P	41	P/Q/E/D	102	0.975	S100 EF-Hand
		V	44	V/L/Q/S/K/N	105	0.993	S100 EF-Hand
		N	45	N/T/K/R/A	106	0.995	S100 EF-Hand
		K	79	K/N/G/Q	140	0.978	S100 EF-Hand
		N	82	N/C/E/D/S	143	0.971	S100 EF-Hand
		I	93	I/L/T/V/I	154	0.987	S100 EF-Hand
		I	95	I/K/D/S/V/K	156	1.00	S100 EF-Hand
		K	99	K/L/N	160	0.992	S100 EF-Hand
		I	100	I/Q/L	161	0.965	S100 EF-Hand
		G	103	G/H/R	164	0.954	
		A	105	A/R/V/E/P/L	166	0.998	
		P	106	P/Q/L/C	167	0.975	
		G	110	G/P/E/H/N	176	0.995	
	*S100A3*	C	83	C/A/V	103	0.952	S100 EF-Hand
		S	95	S/D/P/Q	115	0.957	
**M1a vs. M2a (HGC clade)**	*FLG*	E	3451	K/Q/E/D/R	3690	0.977	
	*FLG2*	G	31	G/D/K/N/R	31	0.954	S100 EF-Hand
		**L**	**41**	**F**	**41**	**0.994**	S100 EF-Hand
		V	44	I/V	44	0.974	
		N	47	N/E/R	47	0.998	
		D	70	D/N	70	0.994	
	*S100A8*	D	22	D/E	61	0.987	

*Human-specific site are indicated in bold face.*

Site-specific positive selection in the more recent great ape HGC clade was identified in a new set of genes; *FLG* [one site], *FLG2* [five sites], and *S100A8* [one site] (Posterior Prob ≥ 0.95) (**Table 4**). Two of the five sites in *FLG2* (codons 31 and 41) were found within the S100 EF-hand domain. We address additional significance of codon 41 below. By contrast, the sites in *FLG* and *S100A8* did not overlap any known conserved domains for these genes but does not necessarily preclude the functional impact of these sites. Together with the 14-mammal and primate specific BS-LRTs, the data suggests site-specific positive selection in *SPRR4* across all 14 mammals as well as episodic positive selection for *KPRP, S100A11, S100A3, S100A7L2*, and *SPRR1A* in primates, and very recent positive selection for *FLG, FLG2*, and *S100A8* in the great ape clade.

### The BS-LRT Identifies Human-Specific Positive Selection in FLG2 and is Found in Neanderthal But Not Denisova

We next sought to identify evidence of positive selection specific to humans. To do this, we examined our BS-LRT results for evidence of human and site-specific positive selection. Human-specific substitutions were only observed in *FLG2* in the HGC test using the M1a–M2a comparison [L41, (F)TTT→(L)CTT] (L41, Posterior Prob. *=* 0.994, respectively) (**Figure 3**; **Table 4**). Further investigation revealed a common SNP (rs3818831, 121T > C transition, with C as the major allele in modern humans) underlying the L41 substitution with an observed major allele frequency of 0.63 (Sherry et al., 2001). L41 occurs close to a cluster of calcium binding sites in the S100 EF-hand domain of *FLG2*, suggesting a possible role for this variant in a *FLG2*-mediated response to calcium.

**FIGURE 4:**
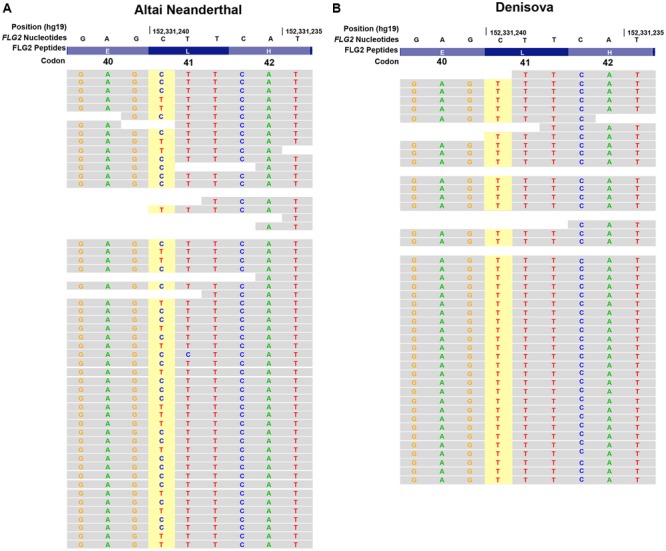
**Human L41 in *FLG2* is found in Altai Neanderthal but not Denisova.** UCSC Genome Browser tracks for short-read alignments of **(A)** Altai Neanderthal and **(B)** Denisova genome reads to hg19 (top track) for *FLG2* L41 (indicated in dark blue). Yellow highlighted area indicates the variant underlying the L41 site identified in the BS-LRT.

We next addressed the origins of the human-specific substitutions in *FLG2.* We determined whether the variants in support of the human-specific residues identified by the BS-LRT arose in the ancient hominids, the Denisova and Neanderthals. The common ancestor of Neanderthals and Denisova diverged from modern humans 700,000–800,000 years ago, while the Denisova lineage diverged from Neanderthals approximately 600,000 years ago (Green et al., 2010; Meyer et al., 2012). Together, the Neanderthals and Denisova represent two separate ancient human lineages with evidence of detectable gene flow events with each other and with modern humans (Green et al., 2010). Closer inspection of the L41 substitution in *FLG2* indicates that the modern allele (Derived Allele: L41, Ancestral Allele: F41) occurred following the split between humans and non-human primates (**Figure 4**). The variant underlying L41 was observed in Neanderthal but not Denisova orthologous sequences (Neanderthal coverage: Total = 48 reads, 19 T [Ancestral], 29 C [Derived]; Denisova coverage: Total = 41 reads, 41 T [Ancestral], 0 C [Derived], **Figures 4A,B**). Thus, the alignments indicate that L41 appeared early in human evolution but has not yet reached fixation in modern humans.

## Discussion

Our results identify the genes and their variants in the EDC locus that are undergoing positive selection across mammalian phylogeny and specific to primates and human. Using both B-LRT and BS-LRT and GA-branch tests, we find significance for more non-synonymous vs. synonymous changes in *FLG, SPRR4, LELP1*, and *S100A2* across 14 mammals. Using foreground clade-specific analyses to determine more recent episodes of positive selection, we further identify positive selection in *SPRR4* that was specific to the primate lineage. GA-branch tests implicated all tested EDC genes except for S100A4 to have undergone positive selection in at least one mammalian node. Furthermore, the GA-branch test further resolved lineage-specific evolution across the mammalian nodes and highlighted *FLG* and *TCHHL1* as the top two genes to evolve across multiple mammalian lineages. Positive selections for *SPRR4, LELP1*, and *S100A2* among many mammalian nodes in the GA-branch test further supports the B-LRT finding. Using the BS-LRT, we also determined site-specific positive selection in *SPRR4, LELP1, FLG*, and *RPTN* across mammalian phylogeny. Recent evolution at specific sites in primates were also found in *S100A11, KPRP, SPRR4, SPRR1A, S100A7L2*, and *S100A3* and *FLG, FLG2*, and *S100A8* in great apes. More recent positive selection was identified in a human-specific *FLG2* variant (L41) in modern humans that was also found in Neanderthal. Together, our study finds positive selection in a diverse set of key EDC genes thus highlighting recent evolution of epidermal skin barrier function in mammalian and human skins.

Our focused study identifying positively selected genes in the EDC in mammals, primates, and human contributes to our understanding of mammalian evolution. Previous genome-wide scans in search of genes undergoing positive selection specifically in humans have implicated several EDC genes in their analyses (Clark and Kosiol papers). Using genome-wide comparisons of human-chimpanzee-mouse genes, Clark et al. (2003) investigated members of the S100 cluster but did not detect significance in their likelihood ratio test. Kosiol et al. (2008) improved on the analysis to identify positively selected genes by using a deeper phylogenetic data set, consisting of three primates (Human, Chimpanzee, Macaque) and four non-primates (mouse, rat, dog, and opossum). They performed likelihood ratio tests on the entire tree, and then on the primate branch to identify episodes of positive selection. In their data set, they identified *SPRR3, LOR*, and *SPRR4* (*p* = 6.64 × 10^-3^, 6.44 × 10^-3^, and 3.32 × 10^-3^, respectively) as being under positive selection.

*SPRR4* and *LELP1* belong to the *SPRR* gene family. SPRR (or small proline rich region) proteins are expressed in the terminally differentiated upper layers of cornified epithelia and at low levels in the cervix and the esophagus (Cabral et al., 2001b). SPRR proteins function as substrates for transglutaminase that crosslinks many EDC proteins together during the formation of the keratinocyte’s cornified envelope for the skin barrier. *LELP1* is both expressed in the epidermis and although their exact functions remain unknown, genetic *LELP1* variation was associated with high IgE levels in humans (Sharma et al., 2007). Site-specific positive selection in *LELP1* sites in the conserved cysteine and proline rich domain highlight the evolving biochemical properties of this novel SPRR protein. Our identification of positive selection in *SPRR4* evolution further validates the protein diversification of these genes that belong to the group 1 *SPRR* cluster (Cabral et al., 2001b). *SPRR4* is highly expressed in the human stratum corneum upon exposure to UV radiation and further supports an adaptive role for *SPRR4* (Cabral et al., 2001a,b; Henry et al., 2012). The BS-LRT enabled us to further determine the molecular evolution of *SPRR4* with positive selection of codon 60 site in the conserved cornifin domain across mammals in contrast to non-cornifin domain in codons 26, 39, 43, 70, and 78 that were selected in the primate lineage. Together, our genomic findings pinpoint the occurrences of these specific *SPRR* genes in the mammalian lineage with recent selection for biochemical sites outside the cornifin domain suggesting ongoing molecular evolution for *SPRR4*.

Positive selection across mammalian phylogeny was also found in *S100A2*, a member of the S100 family. Many of the *S100* proteins encoded in the EDC are associated with calcium signal transduction and are expressed in the granular layer of the epidermis (Eckert et al., 2004; Glaser et al., 2005). Although antimicrobial activity has not been demonstrated for *S100A2*, the paralogy to *S100A9* of known AMP activity (Brandtzaeg et al., 1995; Clark et al., 2016) suggests that *S100A2* may also exhibit AMP activity as well.

Finally, we also observed positive selection in *FLG* across our 14-mammal study and human-specific site selection for L41 in *FLG2*. Both genes belong to the *FLG*-like or SFTP fused domain family whose members possess both a fused S100 domain and an EF hand domain (Wu et al., 2009). *FLG* is a key structural protein in the epidermis that aggregates with keratin filaments (Sandilands et al., 2009; Brown and McLean, 2012). Initially a profilaggrin precursor, FLG is post-translationally cleaved to single filaggrin monomers that metabolically contribute to the natural moisturizing factor of the skin. Like *FLG, FLG2* is also expressed in the differentiated granular layer of the epidermis and is proteolytically degraded (Hsu et al., 2011). The observance of the *FLG2* L41 substitution in ancient hominids suggests that an additional episode of positive selection led to changes in the epidermal barrier integrity in modern humans and has not reached fixation in modern humans. We speculate that the phenotype associated with this positive selection may have been a fitness advantage in the context of dry arid environments during human migration in Eastern and Southern Africa (Carrier et al., 1984). To extrapolate on filaggrin evolution, interestingly, loss-of-function (LOF) mutations for *FLG* are strong risk factors for atopic dermatitis, a common inflammatory skin disease (Sandilands et al., 2006; Brown et al., 2008; Irvine et al., 2011). The allele frequencies for *FLG* LOF of European descent (specifically, Irish) are common, approximately 10% (Sandilands et al., 2006, 2007). Moreover, while LOF mutations in *FLG* are widely replicated across many populations for AD susceptibility, the LOF mutations are inherently unique to each ethnically distinct population that has been studied (in other words, no two *FLG* LOF mutations are alike). Together, the observations suggest recent and independently parallel emergences of potentially positively selected mutations that also converge on AD risk. Similarly, in the absence or rarity of *FLG* LOF mutations in AD patients of African descent, stop gain mutations in *FLG2* instead have been found (Margolis et al., 2014). These observations suggest more recent and parallel selective pressures acting on the evolution for *FLG* and *FLG2* even in the context of disease susceptibility in modern humans. Together, positive selection of the *FLG* epidermal genes highlight mammalian macroevolution and perhaps even more recent human microevolution at the environmental interface for which further biological investigations are warranted.

Together, our results reveal, for the first time, the genetic underpinnings that highlight recent episodic positive selection in epidermal barrier function in mammalian, primate, and modern human skin evolution. This is in contrast to previous studies in search of observable differences in pigmentation and hair follicle density across human populations (Carrier et al., 1984; Elias et al., 2009; Jablonski and Chaplin, 2010). Major changes in human skin have also been reported and associated with habitation of xeric environments characterized by dryness, high temperatures, and high levels of UV-B exposure (Elias et al., 2009). By contrast, genomic scans discovered variations in *EDAR* and *EDA2R* for human hair follicle variation and *SLC24A5* and *SLC45A2* in pigmentation in human skin evolution (Sabeti et al., 2007) and for which the *EDAR V370A* variant was further functionally determined to affect hair thickness as well as a higher density of sweat glands (Kamberov et al., 2013). As much as we have found compelling evidence for positive selection in the skin barrier in modern, ancient humans, and primates, it is likely that the evolution of EDC genes is underestimated. The shared homology within the paralagous members of the *FLG*-like (SFTP), *SPRR, LCE*, and *S100* families in the EDC, including gene fusion in *FLG*-like genes, provides further evidence of the evolution and innovation of the EDC arising via gene duplication and repeat expansion but has been difficult to glean from short sequencing reads and downstream alignments (Cabral et al., 2001b; Strasser et al., 2014). Current sequencing strategies also could have contributed to the lack of fully assessing the evolution of members of the *LCE* (Late Cornified Envelope) gene family (one exon, average 350 bp coding length) that were tested but did not reach significance in our analyses. Furthermore, structural variation including copy number variation, gene duplication, and tandem repeat expansions have been known to contribute to both primate and human evolution (Teumer and Green, 1989; Dumas et al., 2007; Vanhoutteghem et al., 2008; Conrad et al., 2010). It is clear that we need more comparative genomic analyses to better understand the historical events that have shaped skin barrier function and the selection for these genetic variants. In doing so, we will be better equipped to interpret contemporary variation as it pertains to modern disease. Nevertheless, future experiments will address the functional impact for the variants underlying positive selection in the EDC.

## Author Contributions

ZG performed all the experiments. ZG and CdGS did the analyses and wrote the paper.

## Conflict of Interest Statement

The authors declare that the research was conducted in the absence of any commercial or financial relationships that could be construed as a potential conflict of interest.
